# Cell Migration in 1D and 2D Nanofiber Microenvironments

**DOI:** 10.1007/s10439-017-1958-6

**Published:** 2017-11-17

**Authors:** Horacio M. Estabridis, Aniket Jana, Amrinder Nain, David J. Odde

**Affiliations:** 1 Department of Biomedical Engineering, University of Minnesota, 312 Church St. SE, 7-132 Nils-Hasselmo Hall, Minneapolis, MN 55455 USA; 2 Department of Mechanical Engineering, Virginia Tech, Blacksburg, VA 24061 USA

**Keywords:** Migration, Simulation, Parameterization, Persistent random walk, Random walk, Position, Displacement, Glioblastoma

## Abstract

Understanding how cells migrate in fibrous environments is important in wound healing, immune function, and cancer progression. A key question is how fiber orientation and network geometry influence cell movement. Here we describe a quantitative, modeling-based approach toward identifying the mechanisms by which cells migrate in fibrous geometries having well controlled orientation. Specifically, U251 glioblastoma cells were seeded onto non-electrospinning Spinneret based tunable engineering parameters fiber substrates that consist of networks of suspended 400 nm diameter nanofibers. Cells were classified based on the local fiber geometry and cell migration dynamics observed by light microscopy. Cells were found in three distinct geometries: adhering two a single fiber, adhering to two parallel fibers, and adhering to a network of orthogonal fibers. Cells adhering to a single fiber or two parallel fibers can only move in one dimension along the fiber axis, whereas cells on a network of orthogonal fibers can move in two dimensions. We found that cells move faster and more persistently in 1D geometries than in 2D, with cell migration being faster on parallel fibers than on single fibers. To explain these behaviors mechanistically, we simulated cell migration in the three different geometries using a motor-clutch based model for cell traction forces. Using nearly identical parameter sets for each of the three cases, we found that the simulated cells naturally replicated the reduced migration in 2D relative to 1D geometries. In addition, the modestly faster 1D migration on parallel fibers relative to single fibers was captured using a correspondingly modest increase in the number of clutches to reflect increased surface area of adhesion on parallel fibers. Overall, the integrated modeling and experimental analysis shows that cell migration in response to varying fibrous geometries can be explained by a simple mechanical readout of geometry *via* a motor-clutch mechanism.

**Electronic supplementary material:**

The online version of this article (10.1007/s10439-017-1958-6) contains supplementary material, which is available to authorized users.

## Introduction

Cell migration is important in a wide range of settings, including wound healing and cancer progression.^7^ For example, glioblastoma is an aggressive form of cancer that can quickly spread throughout a patient’s brain using microstructural pathways, like axon tracts and blood vessels.^3^ Understanding these avenues for migration of glioblastoma cells through the brain represents an aspect of the disease that could potentially be targeted by new treatment options. Development of an *in vitro* system, and a computational model that explains behavior in it, could elucidate migration mechanisms and aid in the development of potential treatment strategies for processes that rely on cell migration along defined structures.

**Figure 1:**
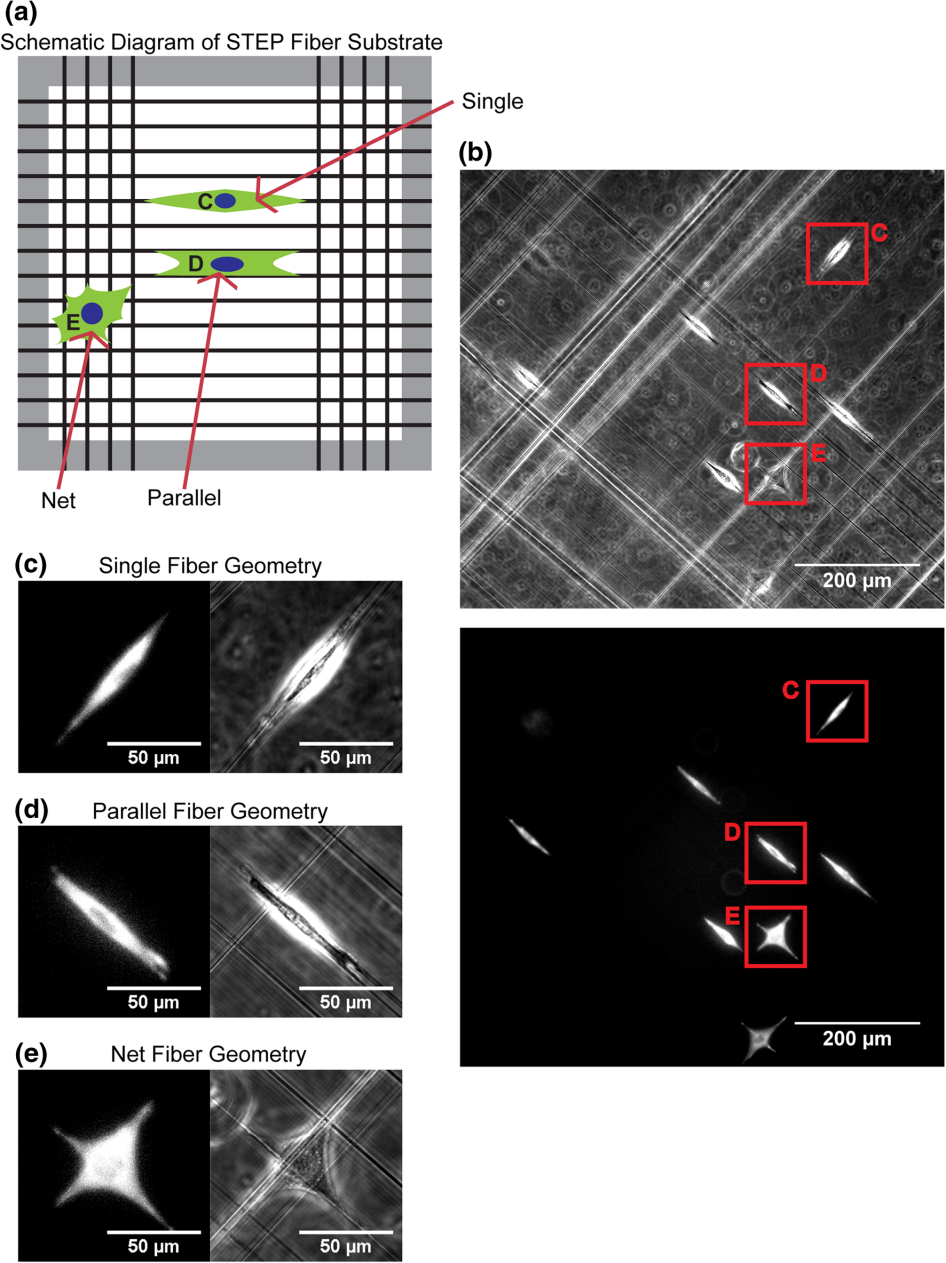
Experimental setup and description of the three geometries encountered by U251 cells. **(A)** A schematic cartoon diagram of the STEP fiber substrate. Cells in the three different geometric environments are labeled **C**, **D** and **E**. **(B)** GFP (top) and phase contrast (bottom) image of U251 GFP-Actin expressing cells seeded onto STEP Fiber substrates. Cells were imaged for 5 h at fifteen minute intervals. Red boxes identify the three different geometries that cells encounter **C**,**D** and **E**. **(C)** GFP (L) and phase contrast (R) image of a cell on a single fiber (region “**C**” from Fig. 1B). **(D)** GFP (L) and phase contrast (R) image of a cell straddling two parallel fibers (region “**D**” from Fig. 1 **B**). **(E)** GFP (L) and phase contrast (R) image of a cell suspended on a fiber network (region “**E**” from Fig. 1 **B**).

Toward this goal, we explored the use of STEP Fibers as a nanoscale system that somewhat replicates the restricted geometry along capillary and axonal structures. STEP Fiber arrays contain within them diverse, complex geometries with ability to control fiber material type, diameter, orientation, and spacing.^18^ Our experiments used substrates with two regions of crossed nanofibers having diameters of approximately 400 nm in a net-like pattern with regions of freely spanning nanofibers in between^18^ (Fig. 1A). STEP Fiber substrates are mechanically anisotropic: though made of amorphous polystyrene (Elastic Modulus = 1–3 GPa) the diameter of the nanofibers is such that cells have the ability to laterally deflect the free span regions. However, cells are not predicted to be able to generate sufficient force to buckle a nanofiber through axial loading, and buckling is not observed experimentally. The combination of geometric variety and anisotropy makes the STEP Fiber substrate distinct from other systems used to study cellular migration, like micro-patterned lanes,^22^ channels,^8^ and 2D surfaces.^14^

Using the DBTRG-05MG glioblastoma cell line, the Nain research group studied blebbing dynamics of cells on STEP Fiber substrates.^21^ They found that cells exhibit three primary morphologies adhering to this substrate: spindle, rectangular and polygonal.^21^ The spindle morphology when cells that were suspended on one single fiber. The rectangular morphology when cells adhered to two parallel fibers. Finally, the polygonal morphology when cells adhered to orthogonal fibers or were in the crosshatched net region of the substrate. The geometry-driven morphology affected the blebbing dynamics of the DBTRG-05MG cells, and appeared to affect the speed the cells migrated.^21^ It is these geometry-driven differences that have motivated the present study and informed the hypothesis that these fibers could replicate brain structures. The study Sharma *et al.* showed that geometry affected cellular behavior in STEP Fibers, it did not explain the mechanisms behind them, which we now address.

In order to develop a mechanistic understanding of Sharma *et al.* results, a stochastic model of cellular migration was developed based on our 2D cell migration simulator.^13^ The cell migration simulator models the action of individual adhesion proteins (termed “clutches”, e.g., integrins or CD44) and myosin motor proteins (termed “motors”).^4^,^13^ The speed of a simulated cell is very sensitive to the ratio of motors to clutches.^2^ Previous work revealed that cell adhesivity effects the speed of glioma cell migration and correlates with CD44-mediated migration.^13^ In this study, both high and low concentrations of CD44 result into lower cell speeds. However, intermediate CD44 concentrations are significantly faster.^13^ Importantly, this study showed that cell migration speed is anti-correlated with mouse and human disease survival. Intermediate adhesivity leads to the fastest migration in mice and the worst survival outcomes in mice and humans.^13^ To use this cell migration simulator to predict cell migration behavior in fibrous environments, we need to incorporate the mechanical features of the nanofibers, such as their stiffness, mechanical anisotropy, orientation, diameter, and spacing. Adding these features to the model allowed us to investigate the mechanisms that drive experimentally observed differences in cellular behavior.

## Results

### Persistent Random Walk and Random Walk Models can be Used to Parameterize Cellular Migratory Behavior in 1D and 2D, Respectively

**Figure 2:**
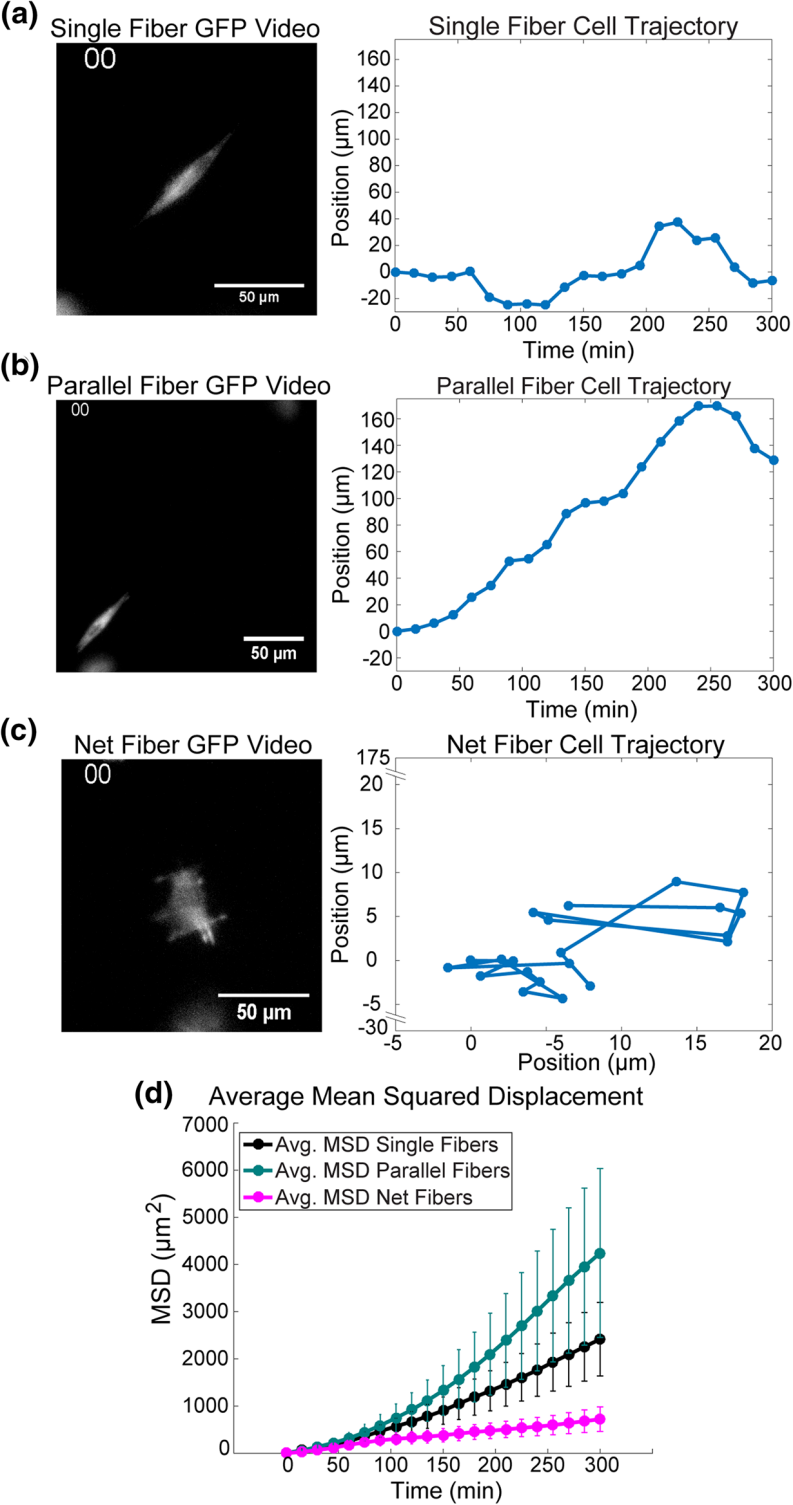
Representative position vs. time plots and average mean squared displacement for each experimental geometry. **(A)** GFP channel video (Supplement) (L) and 1D Position-vs.-time plot (R) for a cell on a single fiber. **(B)** GFP channel video (Supplement) (L) and 1D Position-vs.-time plot (R) for a cell on two parallel fibers. **(C)** GFP Channel video (300 min) (Supplement) (L) and a 2D position plot (R) for a cell on net fiber structure. **D)** Average Mean Squared Displacement for all cells in each geometry (Single Fiber Geometry (n experiments = 14, *n* cells = 90), Parallel Fiber Geometry (n experiments = 14, *n* cells = 94) and Net Fiber Geometry (*n* experiments = 14, *n* cells = 97)).

**Figure 3:**
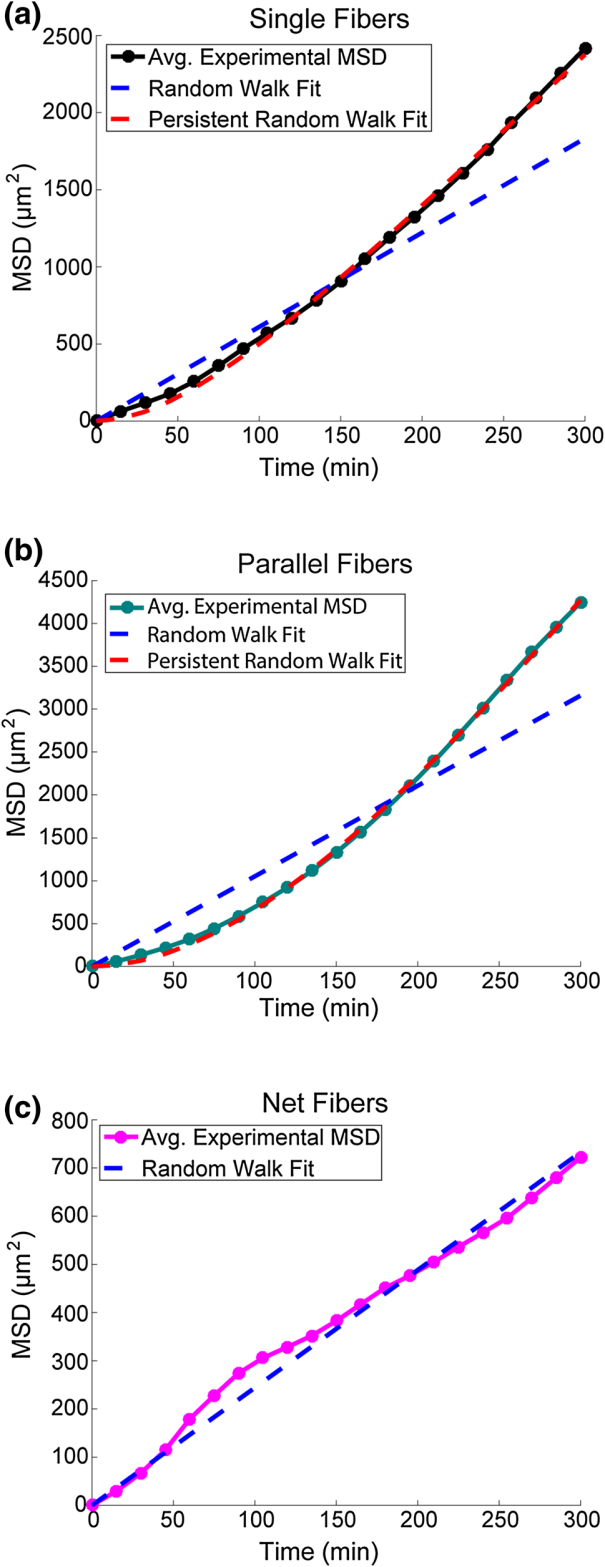
Comparison of persistent random walk and random walk models to experimental average mean squared displacement. **(A)** Comparison of the two models fitting the experimental average mean squared displacement data of the single fiber geometry (*n* experiments = 14, n cells = 90). (Random Walk BIC = 232.9 > Persistent Random Walk BIC = 184.3) **(B)** Comparison of the two models fitting the experimental average mean squared displacement data of the parallel fiber geometry (n experiments = 14, n cells = 94). (Random Walk BIC = 260.6 > Persistent Random Walk BIC = 197.2). **(C)** Random Walk model fit to average mean squared displacement of net fiber geometry (n experiments = 14, n cells = 97). (Random Walk BIC = 138.3 < Persistent Random Walk BIC = 141.2).

To investigate the migratory behavior of GFP-actin U251 cells on STEP fibers, cells were imaged *via* phase contrast and fluorescence microscopy for 5 h at 15 min intervals. The three different geometries observed were: cells on single fibers (*n* = 90), cells spanning two parallel fibers (*n* = 94) and cells on net structures (*n* = 97) (Figs. 1A–1E). Cell centroids were then tracked as cells migrated on the fibrous structures (Figs. 2A–2C). The cells were categorized based on the local geometry of the STEP nanofiber.^21^ During the experiment nanofibers were oriented at random angles in the field of view. As a result, coordinate systems for each cell were rotated to ensure that the nanofibers were oriented along a major axis. The single and parallel fiber geometries that were restricted to 1D motion had their coordinate systems rotated clockwise such that the nanofiber was parallel to the x-axis. The net fibers had their coordinate systems rotated clockwise to ensure that the fibers and their orthogonal partners aligned with either the x-axis or y-axis. Cellular migration is a noisy process that is best expressed using Mean squared displacement (MSD) a calculation that averages the x and y positions of a cell over time, converting the noisy 2D process of cellular migration into a time dependent expression of average cell position from a start point. MSD data can be used to fit models of stochastic cellular motion such as a random walk or persistent random walk.^23^ MSD time series were calculated for each cell in the three geometric conditions from the position data generated by particle tracking. The MSD data was used to characterize and quantify the migration behavior of cells in the three different geometries. Because cells can exhibit persistent behavior when traveling in 1D or oriented environments^9^,^5^ we tested whether a persistent random walk model was appropriate for describing cell trajectories. The same cells do not appear to exhibit this behavior when traveling on the 2D net structures and trajectories more closely resembled a random walk (Figs. 3A–3C). Since our imaging frequency was ∆*t* = 15 min, any persistence values that were less than the 15 min threshold could not be confidently detected by our experiment. In this case the migration model would collapse to a simple random walk.

1 $${\text{MSD}}\left( t \right) = 2nDt$$

2 $${\text{MSD}}\left( t \right) = nS^{2} P^{2} \left( {e^{{\frac{ - t}{P}}} + \frac{t}{P} - 1} \right)$$

Cell migration has been described by a persistent random walk model (Eq. 2) where *n* is the number of dimensions in which motion is occurring (*n* = 1 for single and parallel fibers, *n* = 2 for net structures), *t* is the time, *S* is the speed of the cell and *P* is the persistence time.^24^ A special case of the persistent random walk is the random walk model, given by Eq. (1) when *P* ≪ Δ*t*, where *D* is the random motility coefficient. It is qualitatively clear from the MSD-vs.-time data that cells in the three different geometries exhibit distinct behaviors (Fig. 2D). MSD values for 1D motion (single and parallel fibers) are an order of magnitude higher than the net fibers (Fig. 2D). This is despite the fact that cells moving in 1D have one less degree of freedom available for movement than cells moving in 2D. To parameterize and quantify cell behavior on the three different geometries the MSD-vs.-time data was fit to both of these models (Figs. 3A–3C). We determined that the two parameter persistent random walk model is a more appropriate approximation of single and parallel fiber MSD-vs.-time data sets *via* the Bayesian Information Criterion (BIC). When fitting with a persistent random walk model to the single and parallel fiber geometries an ≈ 20% reduction in BIC was observed for the persistent random walk model as opposed to the random walk model (single fiber ΔBIC = 21%, parallel fiber ΔBIC = 24%). The net fiber geometry did not have a significant improvement for the persistent random walk BIC value over the random walk (ΔBIC = −2%). Additionally, the model’s best-fit persistence time on the net fibers was 30-fold smaller than the 15 min sampling time (*P*_Net_ = 0.4 min). Therefore a random walk model is more appropriate to describe the 2D motion of the net structures (Fig. 3C). Each model was fit to the average MSD-vs.-time data (Figs. 3A–3B). The distribution of parameter values was estimated from the 95% parameter confidence intervals. The increase in cell persistence and MSD magnitude between 1D and 2D motion occurs despite the fewer degrees of freedom cells have when moving on 1D substrates.

### Cells Have a Longer Persistence Time when Adhering to Two Parallel Fibers

**Figure 4:**
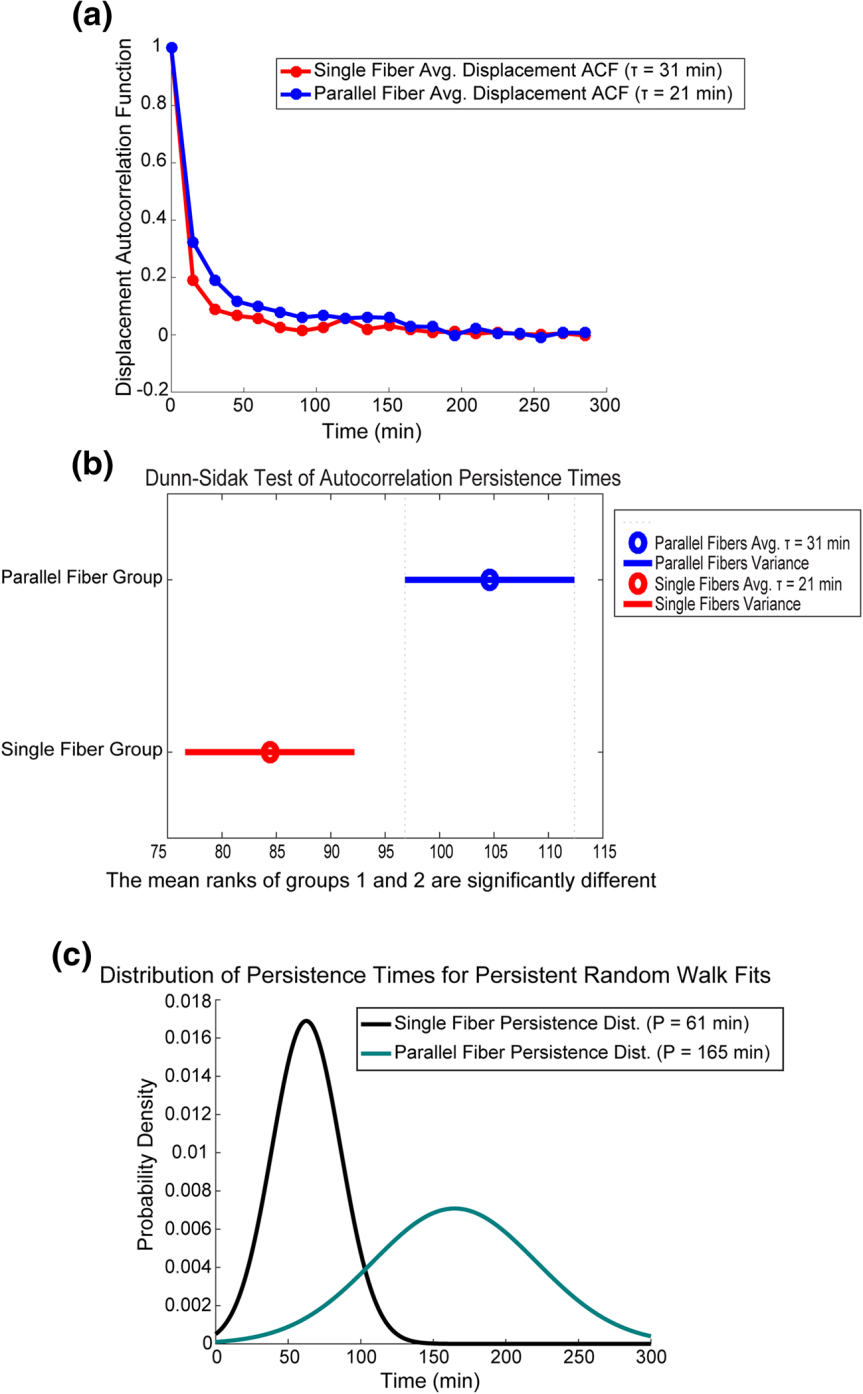
Increased persistence for cell migrating in the parallel fiber geometry. **(A)** Average displacement autocorrelation for cells in single fiber and parallel fiber geometry. Displacement autocorrelation is calculated by finding the displacement between each time point for each cell and performing an autocorrelation.^19^ **(B)** Cells that are seeded onto single fibers or parallel fibers display persistent motion. Cells traveling on two parallel fibers have a longer persistence time than cells that are only on one single fiber. This difference in persistence time is statistically significant (*α* = 0.05) showing that persistent motion is stronger when cells are adherent to two fibers. **(C)** Using parameters extracted from persistent random walk fitting of average behavior of cells we observe that there is a significant difference in persistence times between the single and parallel fiber geometries. Persistence was found to be *μ* = 61 min for single fibers geometry and* μ* = 165 mins for parallel fibers geometry.

The persistence of cells was assessed in two ways: first, the estimated distribution of persistence parameters extracted from the persistent random walk fits were analyzed for a statistically significant shift *via* Student’s *t* test. It was found that the average persistence time of 165 min for the parallel fiber cells was significantly higher than the 61 min average value for the single fiber cells (*P* ≪ 0.01) (Fig. 4C). The second method which was used to analyze the persistent behavior of cells in 1D geometries, was autocorrelation analysis of the displacements of each cell between each sample time point.^19^ Displacements were calculated for each cell in both the single fiber (n = 90) and parallel fiber geometries (*n* = 94). The vector of displacements for each cell was autocorrelated and an exponential decay function was fitted to it (Fig. 4A). The decay time extracted from the autocorrelation fit, represents a persistence time that gives the average amount of time a cell spent traveling in a direction before reversal. It was found that the average of persistence times for parallel fibers (*μ* = 31 min) was again higher than that of the single fibers (*μ* = 21 min) *via* Dunn-Sidak post hoc statistical analysis (*α* = 0.05) (Fig. 3B).

### Stochastic Cell Migration Model Captures Cellular Behavior on STEP Fibers

**Figure 5:**
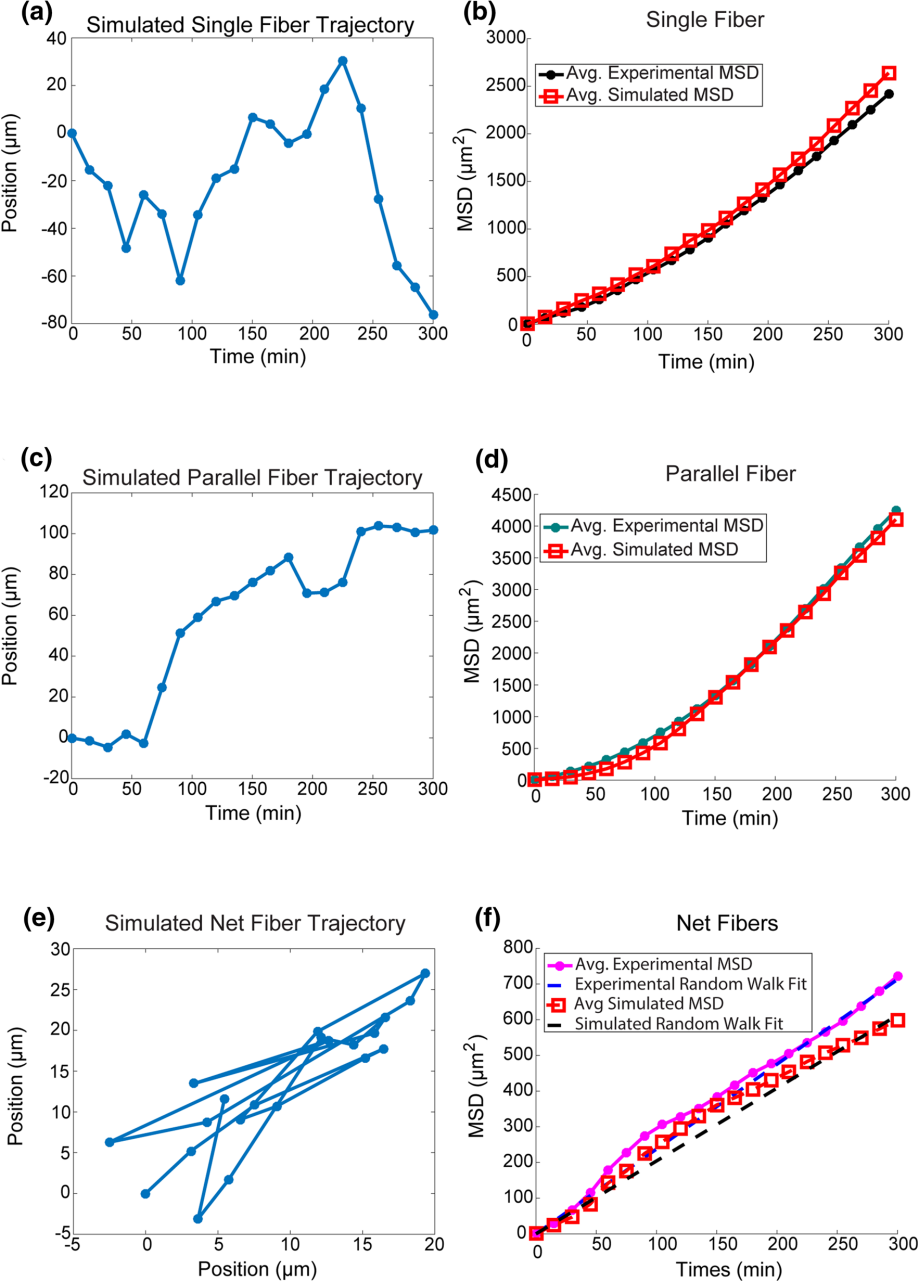
Simulation of cellular migration in a 2D stochastic model of each geometry replicates experimental behavior. **(A)** Trajectory of single fiber simulation. **(B)** Comparison of average experimental and simulated MSD for the single fiber geometry (*n*_simulations_ = 90). **(C)** Trajectory of parallel fiber simulation. **(D)** Comparison of average experimental and simulated MSD for the parallel fiber geometry (*n*_simulations_ = 91). **(E)** Trajectory of net fiber simulation (300 min simulation). **(F)** Comparison of average simulated and experimental MSD for net fiber geometry with average random walk fits to simulation (Avg. RMC = 0.6104 *μ*m^2^/min) and experiment (Avg. RMC = 0.5414 *μ*m^2^/min) (*n*_simulations_ = 91).

In order to better understand the mechanistic basis of the faster migration in 1D vs. 2D, and the higher persistence on parallel fibers vs. single fibers, a stochastic cell simulator was developed to replicate the mechanical environment of the STEP Fiber substrate. Cell migration was simulated using the cell migration model as previously described,^13^ all parameters were identical to Klank *et al.* except with modification to allow for spatial inhomogeneity and mechanical anisotropy in the cellular environment, as described in the methods section. Approximately ninety simulations were run on each geometry to generate a simulated dataset similar in size to the experiments (Single Fibers = 90, Parallel Fibers = 91 and Net Fibers = 91). The simulations were then analyzed using the same procedures as the experiments, and the simulated cells were found to be consistent with the MSD of the different experimental geometries (Figs. 5C–5D, 5F). The simulations also matched the distribution of persistence times extracted from the persistent random walk fit applied to the experimental MSD-vs.-time data of the single $\left( {\mu_{\text{experiment}} = 61 \;{ \hbox{min} },\; \mu_{\text{simulation}} = 62\; { \hbox{min} }} \right)$ and parallel fiber geometries $\left( {\mu_{\text{experiment}} = 165 \;\hbox{min} , \;\mu_{\text{simulation}} = 192 \;\hbox{min} } \right)$. There was a modestly broadened distribution of simulated persistence as opposed to the experimental persistence times (Single Fibers: $\sigma_{\text{experiment}} = 24 \;\hbox{min} , \;\sigma_{\text{simulation}} = 35\; \hbox{min}$, Parallel Fibers: $\sigma_{\text{experiment}} = 56 \;\hbox{min} , \; \,\sigma_{\text{simulation}} = 95 \,\hbox{min}$). The distribution of simulated random motility coefficients is similar to the experimental distribution $\left( {\mu_{\text{experiment}} = 0.6104\;{{\mu {\text{m}}^{2} } \mathord{\left/ {\vphantom {{\mu {\text{m}}^{2} } {\hbox{min} }}} \right. \kern-0pt} {\hbox{min} }} , \mu_{\text{simulation}} = 0.5414\;{{\mu {\text{m}}^{2} } \mathord{\left/ {\vphantom {{\mu {\text{m}}^{2} } {\hbox{min} }}} \right. \kern-0pt} {\hbox{min} }}} \right)$. The simulated single fiber and parallel fiber cells however did not have a statistically significant difference in the persistence time as measured by the displacement autocorrelation function.

Amongst the simulated geometries, the only parameters that varied between simulated cells were the motor-clutch ratios, which was changed exclusively by simply changing the number of clutches. In order to match the experimental behavior the motor-clutch ratios were modified in each geometry. The single fiber geometry had a motor-clutch ratio of 0.926, the parallel fiber geometry had a motor-clutch ratio of 0.735, and the net fiber geometry had a motor-clutch ratio of 1. The difference in motor-clutch ratios in these different geometries leads us to conclude that changes in adhesion is able to explain the differences in cellular behavior in these different geometric conditions. The doubling of potential adhesive surface area for cells spanning two parallel fibers increases the persistence of these cells as the increased number of adhesions reduces the chances of reversing direction. The decrease in the motor-clutch ratio shows that increased adhesion in 1D migration can increase the persistence of this migration. This may explain why cells in channels tend to move persistently.^8^ However, increase in adhesive surface area will eventually hinder cellular migration. A motor-clutch ratio > 1 was found to significantly hinder simulated migration on the two dimensional net fiber geometry.

## Discussion

In this study we investigated the cellular response to well-defined fiber geometries through a combination of experimental analysis and computational modeling using a motor-clutch-based cell migration simulator. Our experimental analysis shows that cells migrating on parallel fibers exhibit increased persistence, as compared to cells traveling on single fibers or two dimensional fiber nets. Qualitatively, our experiments support the conclusion that cells move faster when they are migrating under geometric constraints. Our simulations revealed that an increase in adhesivity in a 1D environment is a potential explanation for this increase in persistence on parallel fibers relative to single fibers. This insight builds on our labs previous work from Reference 13 where we showed that the relative expression levels of CD44 effected patient outcome. Changing the levels of CD44 effects how strongly a cell can bind to the hyaluronic acid rich ECM of the brain. In this work we reinforced the pervious result that intermediate adhesivity leads to the fastest motion. The cells in the parallel fiber geometry had intermediate levels of surface area available to adhesion and moved the fastest. Our results imply that the geometry of the brain influences this phenomenon observed in Reference 13 Cancers with intermediate levels of adhesivity are deadlier because they can effectively leverage the advantages provided by geometric constraints such as axon bundles. The cells do not find themselves bogged down in these structures and can move persistently into new regions of the brain. These results give us quantitative evidence that the speed and persistence of phenomena such as contact guidance^20^ and glioblastoma migration in human brain tissues^1^,^3^,^10^ can be explained simply by both geometry and adhesivity, and does not necessarily require phenotypic or molecular expression changes.

This work was conducted using a simple model of the microfabricated spatially inhomogeneous and anisotropic environments, we simulated and effectively captured the migration behavior of the three different fiber geometries. This was achieved without making any functional changes to the model presented Reference 2. This result shows ability of the motor-clutch model of cellular migration to explain cellular migration in a variety of geometric and mechanical contexts.

### Limitations of Study

While non-electrospun STEP Fibers provide a great number of experimental advantages such as the ability to study cellular migration in a complex yet well characterized mechanical structure it does have some disadvantages. The quality of our images was significantly reduced due to the suspended nature of the STEP fibers. The fibers are suspended approximately 0.8 mm above the surface of glass bottom dishes used for the experiments, which necessitated the use of longer working distance lens having relatively low numerical aperture. One solution would be to use an upright microscope and submerge the object lens in the media above the STEP fibers during experimental observation.

Additionally, our experiments had a relatively low sampling frequency with one image taken every 15 min. This allowed us to observe more cells by multiplexing across multiple fields (typically 40) in one experiment at the cost of reducing the temporal resolution of cellular migration. Therefore, our experiments do not provide information regarding cell migration on time scales shorter than the sampling time (< 15 min).

Our simulations, while able to provide good agreement with the experimental data, were limited by their two dimensional nature. Cells in the parallel fiber geometry were shown to have a statistically significant increase in persistence through the displacement autocorrelation analysis. There are features that we could not account for in our simulations such as the three-dimensional spatial distribution of adhesion molecules that could stabilize the cell. Our stochastic model works by concentrating all adhesions in a cellular protrusion to one point in space. There is also a probability built into our model will randomly disengages a protrusion, significantly reducing the stability of simulated cellular protrusions as opposed to the experiment. This lack of simulated protrusion stability and adhesion distribution likely is the reason the displacement autocorrelation analysis did not show a significant difference in the simulated cells.

### STEP Fibers as a Potential *In Vitro* Mimic of Tissue Microstructures

The non-electrospun STEP fiber substrate has a significant number of mechanical properties that popular 3D culture methods do not have. Culturing cells *in vitro* in collagen, fibrin or Matrigel (Corning Inc., Tewksbury, MA), while potentially more physiologically relevant than 2D culture methods, has difficulty approaching mechanical stiffnesses comparable to *in vivo* tissues. For example, collagen gels have elastic moduli of around 700 Pa.^25^ However, axons and *in vivo* extra cellular matrix (ECM) have been measured to have elastic modulus values of 12,000 and 4000 Pa respectively.^11^ The elastic moduli of ECM is highly heterogeneous spatially and extremely anisotropic. The STEP fibers provide a way to achieve a mechanically well-characterized engineered structure that has the mechanical anisotropy of *in vivo* structures combined with orders of magnitude higher stiffness values. Further development of this technology could potentially yield more accurate *in vitro* tissue mimics that can recapitulate important aspects of the *in vivo* complexity of both geometric and mechanical environments. Perhaps most importantly, the STEP technique permit the fabrication of well-defined fiber geometries. While such patterns might appear to be identical to surface micropatterned lanes on 2D deformable substrates, we not that parallel STEP fibers have mechanical anisotropy, i.e., along the fiber axis vs. transverse across two parallel fibers. However, further work is needed to determine the degree to which mechanical anisotropy influences the behaviors we have observed.

## Materials and Methods

### STEP Fiber Cell Culture

Glioblastoma U251 cells stably expressing GFP-Actin^16^ were cultured in Gibco^®^ Opti-MEM^®^ media (Invitrogen Corporation, Carlsbad, CA, USA) containing 10% fetal bovine serum. Four hundred nm diameter polystyrene STEP Fiber substrates^18^ were attached to MatTek No. 0 glass bottom 35 mm dishes (MatTek Corp., Ashland, MA, USA) onto Dow-Corning^®^ High Vacuum Grease (Dow-Corning Inc., Midland, MI, USA). The STEP Fiber substrate was incubated with 1 mg/mL bovine plasma fibronectin (Sigma-Aldrich Inc., St. Louis, MO, USA) for 4 h at 37 °C prior to cell seeding. 50 μL of cell suspension was seeded onto fibronectin-coated STEP fiber substrates at a concentration of 100,000 cells/mL and incubated for 3 h prior to imaging.

### Cell Migration Imaging

Cells were imaged with a Nikon Instruments Eclipse Ti-E (Nikon Instruments Inc., Melville, NY, USA) for 5 h at 15 min intervals. Widefield phase contrast images were collected with a Nikon 20 × air phase ring objective lens (Nikon Instruments Inc., Melville, NY, USA) and GFP channel images were collected with a Cool LED pE-100 LED fluorescent illumination bank (CoolLED Ltd., Andover, England, UK). Images were taken with an Andor Zyla 5.5 sCMOS camera (Andor Technology Ltd., Belfast, Northern Ireland, UK). Cells were incubated on the microscope through the use of an AirTherm ATX (World Precision Instruments Inc., Sarasota, FL, USA). Image collection was driven by NIS-Elements software (Nikon Instruments Inc., Melville, NY, USA).

### Image Processing and Data Analysis

Cells were classified based on which geometry the cell spent the majority of its time in the time-lapse movie. Wide-field GFP image stacks were cropped to select individual cells and thresholded using ImageJ (National Institutes of Health (NIH), Bethesda, MD, USA). Thresholded image stacks were fed into MATLAB and cell centroids were tracked using software developed in MATLAB 2012a and 2016a (MathWorks Inc., Natick, MA, USA) as described in Reference 13. The cell tracking software works by applying a binary filter to the grayscale images. Using MATLAB’s image processing tool box, objects are labeled and the user selects the cell to track. Cell centroid positions are calculated for each frame for the object the user selects. Cell position data is saved to a plaintext file that can be read back into MATLAB for further processing. Coordinate systems were rotated using MATLAB, which used angles that were calculated in ImageJ. Mean squared displacement was calculated in MATLAB. Random walk and persistent random walk fits performed using the MATLAB Curve Fitting Toolbox. Statistical tests performed using the MATLAB Statistics Toolbox.

### Stochastic Cell Migration Simulator Description

The cell migration simulator works by simulating the action of individual adhesion molecules (termed “clutches”), myosin motor proteins (termed “motors”), and actin subunits, which self-assemble at the leading edge to form simulated cellular protrusions that displace the centroid of simulated cell, as previously described.^13^ Briefly, the total starting amount of each of these three molecular species is fixed, ensuring mass conservation of key species. Upon initialization, cell protrusions extend at randomly chosen angles with a fixed initial length. Each time a new cellular protrusion is nucleated it removes the actin required to form it from the pool of F-actin the cell has initially. Each protrusion is populated with a random number of clutches and motors which is subtracted from the total amount in their respective starting pools. The clutches are able to interact stochastically with the substrate to allow clutch binding and unbinding. Simultaneously, the F-actin in the protrusion is pulled retrograde by the motors, at a velocity given by a linear force–velocity relationship, thus creating a displacement which simulates actin retrograde flow. In order to simulate the well-documented phenomenon of cellular sensitivity to substrate stiffness^6^ the substrate and the clutch are both modeled as linear springs. The clutch springs are arranged in parallel and connected in series with the substrate spring. The F-actin network is modeled as inextensible. The displacement generated by the F-actin retrograde flow generates reaction forces on the clutch and substrate springs. This set of events is occurring in parallel in every protrusion in the cell. Once force is generated, an elastic force balance equation is solved to achieve elastic equilibrium, which assumes that viscoelastic relaxation times are short relative to the time event. This force balance, combined with the random orientation of force-generating protrusions, leads to cellular displacement. The forces generated can also cause protrusions to detach from the substrate: the more force that a clutch experiences, the higher the unbinding rate constant according to Bell’s Law given by the following equation:

3 $$k_{\text{off}} = k_{{{\text{off}},0}} \cdot e^{{\frac{F}{{F_{B} }}}}$$

This sudden protrusion failure will also have the effect of displacing the cell as the forces must rebalance with fewer protrusions. When retrograde flow causes the clutches to pass beyond the myosin motors the protrusion disassembles and the actin, motors and clutches are added back to their respective original pools. Thus performing a mass balance in the simulated system. A new protrusion may then be born with these components again restarting the process. This model of cellular migration has been well-characterized and it has been found that the two most sensitive parameters are the total number of motors and clutches (Table 1).^2^

**Table 1:** Simulation parameter values.

Symbol	Parameter	Single fiber value	Parallel fiber value	Net fiber value	Source
*N*_m_	Total number of motors	125	125	125	Adjustable (estimated based on Ref. 17)
*N*_c_	Total number of clutches	135	170	125	Adjustable (Nm/Nc based on Ref. 2)
*A*_tot_	Total possible actin protrusion length	100 *μ*m	100 *μ*m	100 *μ*m	Typical cell length
*v*_p_*	Maximum actin polymerization velocity	200 nm/s	200 nm/s	200 nm/s	4
*k*_mod_*	Maximum module birth rate	1 s^-1^	1 s^−1^	1 s^−1^	13
*k*_mod_	Typical module birth rate	0.00001 s^−1^	0.00001 s^−1^	0.00001 s^−1^	Must be similar to kcap
*k*_cap_	Module capping rate experiments	0.00001 s^−1^	0.00001 s^−1^	0.00001 s^−1^	Lowered from Ref. 13 to match
*I*_init_	Initial module length	1.5 *μ*m	1.5 *μ*m	1.5 *μ*m	Adjustable
*I*_min_	Minimum module length	0.1 *μ*m	0.1 *μ*m	0.1 *μ*m	Adjustable
*k*_cell_	Cell spring constant	1000 *p*N/nm	1000 *p*N/nm	1000 *p*N/nm	Adjustable
*n*_c,cell_	Number of cell body clutches	1	1	1	Adjustable (< *N*_c_)
*n*_m_*	Maximum number of module motors	25	25	25	Adjustable (0.2*N_m_)
*F*_m_	Motor stall force	2 pN	2 pN	2 pN	17
*v*_m_*	Motor unloaded velocity	120 nm/s	120 nm/s	120 nm/s	4
*n*_c_*	Maximum number of module clutches	27	34	25	Adjustable (0.2*N_c_)
*k*_on_	Clutch on-rate	1 s^−1^	1 s^−1^	1 s^−1^	Increased from Ref. 4
*k*_off_*	Clutch unloaded off-rate	0.1 s^−1^	0.1 s^−1^	0.1 s^−1^	15
*k*_c_	Clutch spring constant	0.8 pN/nm	0.8 pN/nm	0.8 pN/nm	2
*F*_b_	Clutch bond rupture force	2 pN	2 pN	2 pN	12
*k*_sub_	Substrate spring constant	220 pN/nm	220–1 pN/nm	220 pN/nm	Estimated from Euler–Bernoulli beam theory, Eq. (3)

Table contains a breakdown of the parameter values used in stochastic cell migration simulations for each geometry. Sources, justification and derivation of each parameter is outlined

Each geometry was modeled by restricting the region that cells can adhere to on the surface. The single fiber geometry was modeled as a 400 π nm wide lane with uniformly high stiffness (220 *p*N/nm). The parallel fiber geometry was modeled as two parallel 400 π nm wide lanes separated by 4000 nm. The parallel fiber geometry’s mechanical anisotropy was modeled using a model based on Euler–Bernoulli beam theory that depended on the angle of orientation of the protrusion relative to the fiber, θ given by:

4 $$\kappa_{\text{sub}} = \left| {\frac{{\pi Er^{2} }}{L} \cdot \cos \left( \theta \right) + \frac{{12\pi Er^{4} }}{{L^{3} }} \cdot \sin \left( \theta \right) } \right|$$

where *E* is the elastic modulus, *r* is the fiber radius (200 nm), *L* is the fiber length (8 × 10^6^ nm). The first coefficient in the equation $\frac{{\pi Er^{2} }}{L}$ represents the spring constant experienced when the cell is loading the fiber laterally (*θ* = 0°). The second coefficient $\frac{{12\pi Er^{4} }}{{L^{3} }}$ represents the spring constant experienced when the cell is loading the fiber orthogonally (*θ* = 90°). The net fiber geometry was modeled as a series of orthogonal lanes of uniform stiffness (220 *p*N/nm). The gap width between each fiber was identical to the parallel fiber geometry (4000 nm).

## Electronic supplementary material

Below is the link to the electronic supplementary material.

Supplementary material 1 (MOV 263 kb)

Supplementary material 2 (MOV 157 kb)

Supplementary material 3 (MOV 1286 kb)

## Abbreviations

MSD: Mean squared displacement
ACF: Autocorrelation function
STEP: Spinneret tunable engineered parameters
